# Supplementary material for: Not just flowering time: a resurrection approach shows floral attraction traits are changing over time
Source: Evol Lett. 2023 Mar 8;7(2):88–98. doi: 10.1093/evlett/qrad006 (PMC10078973; doi:10.1093/evlett/qrad006)
Supplement: qrad006_suppl_Supplementary_Material [file qrad006_suppl_supplementary_material.pdf]

						Number of Plants			Number of Flowers		Number of Maternal Lines
**ID**	**Population Name**	**Year**	**State**	**Latitude**	**Longitude**	**Morphology**	**Phenology**	**Rewards**	**Morphology**	**Rewards**	**Rewards**
1	billings	2003	TN	35.77524	-85.90342	10	NA	NA	55	NA	NA
1	billings	2012	TN	35.77524	-85.90342	22	NA	NA	191	NA	NA
2	bergaw1	2003	NC	34.59571	-77.92748	13	14	NA	68	NA	NA
2	bergaw1	2012	NC	34.59571	-77.92748	15	2	NA	84	NA	NA
4	chicken road	2003	NC	34.55667	-79.12560	16	12	NA	74	NA	NA
4	chicken road	2012	NC	34.55667	-79.12560	17	12	NA	60	NA	NA
5	clarendon1	2003	SC	33.85988	-79.90907	NA	13	NA	NA	NA	NA
5	clarendon1	2012	SC	33.85988	-79.90907	NA	1	NA	NA	NA	NA
8	darlington2	2003	SC	34.29720	-79.99126	16	10	NA	90	NA	NA
8	darlington2	2012	SC	34.29720	-79.99126	12	11	NA	46	NA	NA
9	duplin east	2003	NC	34.92404	-77.79617	NA	13	26	NA	179	8
9	duplin east	2012	NC	34.92404	-77.79617	NA	4	8	NA	65	6
10	duplin west	2003	NC	34.98316	-78.03931	NA	9	NA	NA	NA	NA
10	duplin west	2012	NC	34.98316	-78.03931	NA	2	NA	NA	NA	NA
11	grimsley	2003	NC	34.52714	-78.75670	NA	5	NA	NA	NA	NA
11	grimsley	2012	NC	34.52714	-78.75670	NA	16	NA	NA	NA	NA

12	florence	2003	SC	34.14581	-79.86531	18	12	28	78	195	8
12	florence	2012	SC	34.14581	-79.86531	13	11	31	83	222	8
14	hare road	2003	NC	35.42476	-77.91712	16	14	32	106	208	8
14	hare road	2012	NC	35.42476	-77.91712	17	11	31	97	213	8
15	horry1	2003	SC	34.10421	-79.07373	15	10	*NA*	94	*NA*	*NA*
15	horry1	2012	SC	34.10421	-79.07373	12	13	*NA*	53	*NA*	*NA*
16	horry2	2003	SC	34.10535	-79.18323	13	9	*NA*	101	*NA*	*NA*
16	horry2	2012	SC	34.10535	-79.18323	29	11	*NA*	177	*NA*	*NA*
17	marion1	2003	SC	34.15915	-79.27291	15	11	*NA*	97	*NA*	*NA*
17	marion1	2012	SC	34.15915	-79.27291	18	12	*NA*	96	*NA*	*NA*
18	marion2	2003	SC	34.15659	-79.27027	*NA*	11	*NA*	*NA*	*NA*	*NA*
18	marion2	2012	SC	34.15659	-79.27027	*NA*	13	*NA*	*NA*	*NA*	*NA*
19	mckinnon	2003	NC	34.50819	-78.70899	17	13	*NA*	135	*NA*	*NA*
19	mckinnon	2012	NC	34.50819	-78.70899	20	8	*NA*	195	*NA*	*NA*
21	new hope	2003	NC	35.36982	-77.87731	*NA*	13	*NA*	*NA*	*NA*	*NA*
21	new hope	2012	NC	35.36982	-77.87731	*NA*	11	*NA*	*NA*	*NA*	*NA*
22	oldkenley	2003	NC	36.14360	-78.05342	18	12	*NA*	125	*NA*	*NA*
22	oldkenley	2012	NC	36.14360	-78.05342	27	10	*NA*	279	*NA*	*NA*
23	snakes	2003	TN	35.06791	-86.62955	*NA*	3	*NA*	*NA*	*NA*	*NA*
23	snakes	2012	TN	35.06791	-86.62955	*NA*	11	*NA*	*NA*	*NA*	*NA*
25	starlight	2003	NC	34.61636	-79.05167	17	12	*NA*	89	*NA*	*NA*
25	starlight	2012	NC	34.61636	-79.05167	1	5	*NA*	4	*NA*	*NA*

26	spears soy	2003	TN	35.53341	-85.95190	NA	3	NA	NA	NA	NA
26	spears soy	2012	TN	35.53341	-85.95190	NA	9	NA	NA	NA	NA
28	sumter2	2003	SC	34.09792	-80.37771	16	9	25	62	187	8
28	sumter2	2012	SC	34.09792	-80.37771	13	9	29	60	199	8
29	tarheal	2003	NC	34.70513	-78.73890	14	12	NA	63	NA	NA
29	tarheal	2012	NC	34.70513	-78.73890	10	9	NA	65	NA	NA
30	willis corn	2003	TN	35.31105	-85.94500	NA	11	NA	NA	NA	NA
30	willis corn	2012	TN	35.31105	-85.94500	NA	9	NA	NA	NA	NA
31	vervilla	2003	TN	35.60848	-85.84638	NA	10	NA	NA	NA	NA
31	vervilla	2012	TN	35.60848	-85.84638	NA	10	NA	NA	NA	NA
32	walnut grove	2003	TN	35.09936	-86.22551	6	NA	NA	57	NA	NA
32	walnut grove	2012	TN	35.09936	-86.22551	10	NA	NA	52	NA	NA

Table S1. Sampling localities included in the phenotypic analyses including geographic coordinates, the number of plants included for each of the three greenhouse experiments, the number of flowers measured for the floral morphology and floral rewards experiments, and the number of maternal lines used in the floral rewards experiment. Abbreviations: TN: Tennesses, NC: North Carolina, SC: South Carolina

		**Corolla Width**	**Corolla Length**	**ASD**	**First Flowering Wave**	**Second Flowering Wave**	**°Brix**	**Pollen Count**
**Trait ~ Year*Latitude + (1|Population)**								
**Year**	numDF	1	1		1	1	1	1
	denDF	12.10	11.77	9.67	289.89	141.09	1.94	1.90
	F	7.093	10.472	4.42	3.950	0.206	0.003	0.028
	p	0.0205**	0.007**	0.659	0.048**	0.651	0.961	0.883
**Latitude**	numDF	1	1	1	1	1	1	1
	denDF	2662.85	2781.94	2468.51	20.98	22.10	200.63	163.41
	F	16.850	0.041	1.587	0.121	5.484	1.94	2.187
	p	4.167e-05**	0.840	0.072	0.731	0.029**	0.016**	0.141
**Year*Latitude**	numDF	1	1	1	1	1	1	1
	denDF	519.82	923.79	1576.65	290.00	141.16	60.45	33.25
	F	23.388	0.580	2.633	3.950	0.203	4.59	2.180

	p	1.747e-06**	0.447	0.641	0.048**	0.653	0.036**	0.149
***δt* ~ t*Latitude**								
**t**	numDF	1	1	1	1	1	NA	NA
	denDF	11	11	11	19	16	NA	NA
	F	2.600	3.507	26.905	38.307	6.291	NA	NA
	p	0.14	0.088*	3.004e-4**	6.022e-6**	0.023**	NA	NA
**Latitude**	numDF	1	1	1	1	1	NA	NA
	denDF	11	11	11	19	16	NA	NA
	F	2.654	1.277	3.055	0.789	1.157	NA	NA
	p	0.13	0.283	0.108	0.386	0.298	NA	NA
**t*Latitude**	numDF	1	1	1	1	1	NA	NA
	denDF	11	11	11	19	16	NA	NA
	F	6.058	0.671	0.345	3e-4	0.792	NA	NA

	p	0.032**	0.430	0.569	0.987	0.387	NA	NA

Table S2. Linear mixed models showing the influence of latitude on temporal changes in trait value and the predictability of *δt* by *t*. The first model shows significance of latitude, sampling year, and the interaction of the two on all trait values, reported as a truncated p-value based on Satterwaite's degrees of freedom method for a type III ANOVA. For floral morphology and phenology, latitude is included as a fixed effect while population is used as a random effect to control for longitude. The model for floral rewards additionally uses maternal line as a nested random effect within population (Trait ~ Year*Latitude + (1|Pop/ML)). The second model shows the interactive effect of latitude and starting population mean trait value on the degree of change in mean trait value for a population. Significance (p<0.05) is marked with a double asterisk** and relationships trending significant (0.05 < p < 0.1) are marked with a single asterisk*.

	**Year**	**Range**	**Mean**	**SD**	**PCV (%)**	**% Change PCV**	**Significance (p)**
**Corolla Width**	2003	1.8-7.5	4.503	0.993	21.976	NA	NA
	2012	1-7.1	4.764	0.993	20.849	5.406	0.063*
**Corolla Length**	2003	2.8-8	5.431	0.616	11.463	NA	NA
	2012	2.2-7	5.474	0.6165	11.259	1.812	0.642
**ASD**	2003	-2.7-1.3	0.116	0.256	212.296	NA	NA
	2012	-1-1.15	0.108	0.201	186.923	13.57	0.387

**Flowering Wave 1**	2003	213-240	223.2	8.112	3.629	NA	NA
	2012	214-241	223	7.466	3.343	8.555	0.211
**Flowering Wave 2**	2003	256-305	277.8	13.82	4.959	NA	NA
	2012	257-307	278.3	14.26	5.103	-2.822	0.753
**Nectar Sucrose Content**	2003	0-21	6.524	3.056	46.838	NA	NA
	2012	0-22	6.805	2.903	42.66	9.794	0.048**
**Pollen Count**	2003	3-508	191.5	59.25	30.925	NA	NA
	2012	2-493	200.8	49.92	24.855	24.42	0.016**

Table S3. Range, least squares mean, and standard deviation of floral trait values are shown for each year. Floral morphology traits are measured in centimeters, flowering dates use a Julian calendar, nectar sucrose content is measured as °Brix, and pollen count is a total number of pollen grains found on a single anther. Also shown is the phenotypic coefficient of variation (PCV) determined using a bootstrapped resampling procedure with 10000 draws, percent change of PCV from 2003 to 2012, and the p-value associated with a two-sided independent t-test comparing levels of phenotypic variation in 2003 to 2012 for each trait. Significance ($p<0.05$) is marked with a double asterisk** and relationships trending significant ($0.05 < p < 0.1$) are marked with a single asterisk*.

	CW:CL		CW:ASD		CL:ASD		CW:FF		CL:FF		ASD:FF	
	2003	2012	2003	2012	2003	2012	2003	2012	2003	2012	2003	2012
Correlation Coefficient (r)	0.611	0.596	0.035	-0.030	-0.055	-0.129	-0.237	-0.008	-0.188	0.081	0.364	-0.174
Significance (p)	<2.2e-16**	<2.2e-16**	0.223	0.253	0.047**	3.681e-07**	0.245	0.970	0.359	0.695	0.068*	0.394

Table S4. Pearson's correlation coefficients and associated p-values for floral morphology traits and phenology. Brix and pollen count excluded due to low population sampling. Since floral traits and phenology are not paired data, correlation is calculated with population means, otherwise correlation coefficients and p-values are calculated from raw measurements. Repeating a Pearson's correlation test on floral traits using population means yields a higher correlation between floral traits. Abbreviations: CW = Corolla Width, CL = Corolla Length, ASD = Anther Stigma Distance, FF = date of first flower. Significance ($p<0.05$) is marked with a double asterisk** and relationships trending significant ($0.05 < p < 0.1$) are marked with a single asterisk*.
